# Current Evidence for a New Surgical Technique for Scleral Fixation: The Implantation of a Carlevale Lens, a Systematic Review

**DOI:** 10.3390/jcm13113287

**Published:** 2024-06-03

**Authors:** Francesca Barbieri, Maria Novella Maglionico, Giamberto Casini, Gianluca Guidi, Michele Figus, Chiara Posarelli

**Affiliations:** 1 Department of Surgical, Medical, Molecular Pathology and Critical Care Medicine, University of Pisa, 56126 Pisa, Italy; francesca1.barbieri1@gmail.com (F.B.); marianovella.maglionico@phd.unipi.it (M.N.M.); michele.figus@unipi.it (M.F.); 2 Ophthalmology, Department of Medical and Surgical Specialties, Azienda Ospedaliero Universitaria Pisana, 56124 Pisa, Italy; giamberto.casini@gmail.com (G.C.); gianlu.guidi@gmail.com (G.G.)

**Keywords:** Carlevale IOL, scleral fixation, cataract surgery

## Abstract

**Background**: The Carlevale lens (FIL SSF, Soleko IOL Division, Italy) is a new lens for suture-less scleral fixation. This paper aimed to systematically review articles on this lens, the surgical techniques used for its implantation, complications and outcomes. **Methods**: This systematic review was performed following the PRISMA guidelines. The search string used was “Carlevale” AND “scleral fixation” from inception until March 2024. For completeness, either case-control studies, case reports or case series written in English were included. The authors used the Newcastle–Ottawa scale for the case-control studies and the JBI Critical Appraisal Checklist for case reports and case series. **Results**: Twenty-nine articles were included. Scleral fixation with Carlevale lens can be performed by creating scleral flaps or, alternatively, by using scleral pockets. The two sclerotomies must be diametrically opposed, and are preferably created by 25-gauge trocars. A pars plana vitrectomy should be performed every time, and the design of the lens should be suitable for self-anchoring to the sclera; the most accredited strategy to achieve this is to avoid scleral sutures. There were only a few intraoperative and postoperative complications reported; vitreous hemorrhages were the most frequent intraoperative events, while the most relevant postoperative complications were vitreous hemorrhages, cystoid macular oedema and transient variations in the intraocular pressure. Excellent results have been obtained in terms of BCVA, IOL centration and stability, mean intraocular pressure, postoperative spherical equivalent, separation between anterior and posterior chamber and the distance of the IOL from anterior chamber structures. **Conclusions**: The foldable hydrophilic design of the Carlevale lens has shown good effectiveness, IOL stability and few intra and post-operative complications.

## 1. Introduction

The gold standard technique for cataract surgery is the implantation of an intraocular lens (IOL) in the bag, ensuring both stability and optimal lens placement [1]. However, in the absence of capsular support or an inadequate posterior bag, surgeons face the challenge of finding the best solution for IOL implantation.

Surgical options for IOL implantation include anterior chamber IOLs (ACIOLs), iris-fixated IOLs (IFIOLs) for the anterior chambers and scleral-fixated IOLs (SFIOLs) for the posterior chamber [2,3]. ACIOLs and IFIOLs require large incisions for implantation, which result in substantial postoperative astigmatism. ACIOLs are often associated with complications such as a significant induced astigmatism, bullous keratopathy and increased intraocular pressure [4,5]. IFIOLs can be placed either anterior or posterior to the iris without sutures, but they require an intact iris diaphragm [6]. Additionally, these lenses can damage the pupil and iris, and lead to a loss of endothelial cells [6,7].

For these reasons, scleral-fixated IOLs represent a better solution. As reported by Fiore and colleagues, positioning the IOL away from anterior segment structures minimizes the incidence of adverse events such as glaucoma and bullous keratopathy [8,9].

Maggi and Maggi first described scleral fixation through the transscleral passage of the haptics and, over the time, the surgical technique has undergone several modifications [10].

Regarding fixated IOLs (SFIOLs), we know they can be divided into scleral sutured IOLs and suture-less ones [8]. In both cases, until recently, the reference IOLs were three-piece IOLs. Unfortunately, the design of the three-piece IOL, combined with the anatomical differences in the eyes, is often responsible for the incorrect centering of the IOL and the position of the haptic in contact with the iris and other anterior segment structures. Moreover, in case of transscleral suturing IOLs, the degradation of the suture material may increase the risk of suture erosion and associated endophatlmitis, lens tilt and dislocation [11]. 

In light of this evidence, further research has aimed to investigate new suture-less surgical strategies. Agarwal proposed the use of glue for scleral fixation [12], while Yamane and colleagues created two angled sclerotomies and proposed cauterizing the haptics to create a flange [13]. 

To address issues related to three-piece IOLs, modifications to the intraocular lens were considered. Consequently, an intraocular one-piece acrylic lens called the FIL-SSF Carlevale lens (Soleko IOL Division, Pontecorvo, Italy) has been introduced [8].

The aim of this study is to systematically review the literature and evidence about this new lens; to identify the suggested surgical technique of implantation; and to report visual outcomes, and intra and postoperative complications. This information will help surgeons better understand the lens and achieve optimal postoperative outcomes.

## 2. Materials and Methods

This is a systematic review that aims to describe the advent of Carlevale lens, the surgical techniques involved in lens implantation and the relative outcomes. The systematic approach of a bibliographic search and the selection of studies followed PRISMA guidelines [14].

### 2.1. Data Sources and Search Strategy

A literature search was conducted using the following keywords: “Carlevale” AND “scleral fixation” from their introduction until March 2024.

The search was conducted on MEDLINE (OVID and PubMed), Google Scholar, ISI Web of Science (Thom-on-Reutets), Cochrane Library (Wiley) and Scopus databases until March 2024. Two investigators (F.B and M.N.M) independently examined the titles and abstracts. The investigators also performed a further search for additional studies; the search was performed among the reference of the articles. 

### 2.2. Selection and Eligibility 

The selection of studies followed a three-step approach: (a) studies regarding the design of the Carlevale lens; (b) studies describing the surgical technique; (c) studies describing positive and/or negative outcomes. Since there is a lack of randomized trials in the literature, for completeness, case series, case control studies and case reports published in English language and in peer-reviewed journals were also included. Study location did not represent a restriction. The following articles were excluded: systematic and narrative reviews, randomized controlled trials, meta-analysis, studies conducted on animals and in a language other than English. During the screening, three articles were also excluded because they did not report data about either the surgical technique or complications or visual outcomes. One article was also excluded because it was a description of a surgical technique, but the risk of bias was not assessable.

### 2.3. Data Extraction

Two investigators (F.B and M.N.M) independently examined each article.

From each examined study we extracted the following data: last name of the first author, year of publication, study design, purpose, surgical technique, intraoperative and postoperative complications, visual outcome and conclusions.

During the bibliographic selection, meta-analysis studies, systematic and narrative reviews were excluded; randomized controlled trials were not present in the literature. Once the eligible studies have been identified, the investigators checked their references to find other suitable studies.

### 2.4. Risk of Bias and Quality of Evidence Assessment

Two investigators (F.B and M.N.M) independently assessed the risk of bias by using the Joanna Briggs Institute (JBI) Critical Appraisal Checklist for Case Series/case reports and the Newcastle–Ottawa scale for case control studies [15]. The authors assessed the following thresholds for converting the Newcastle–Ottawa scales: good quality = above 6 stars; fair quality = 4 to 6 stars; poor quality = 0 to 3 stars [16]. Since there is a lack of randomized trials in the literature, in this review it was not possible to evaluate the Quality of Evidence by applying the Grading of Recommendations Assessment, Development and Evaluation (GRADE) system [17] or by awarding a “Level” and “Strength” to the examined works [18,19].

Risk of Bias and Quality assessments are displayed in Supplementary Materials (Tables S1–S3).

## 3. Results

### 3.1. Search Results 

**Figure 1:**
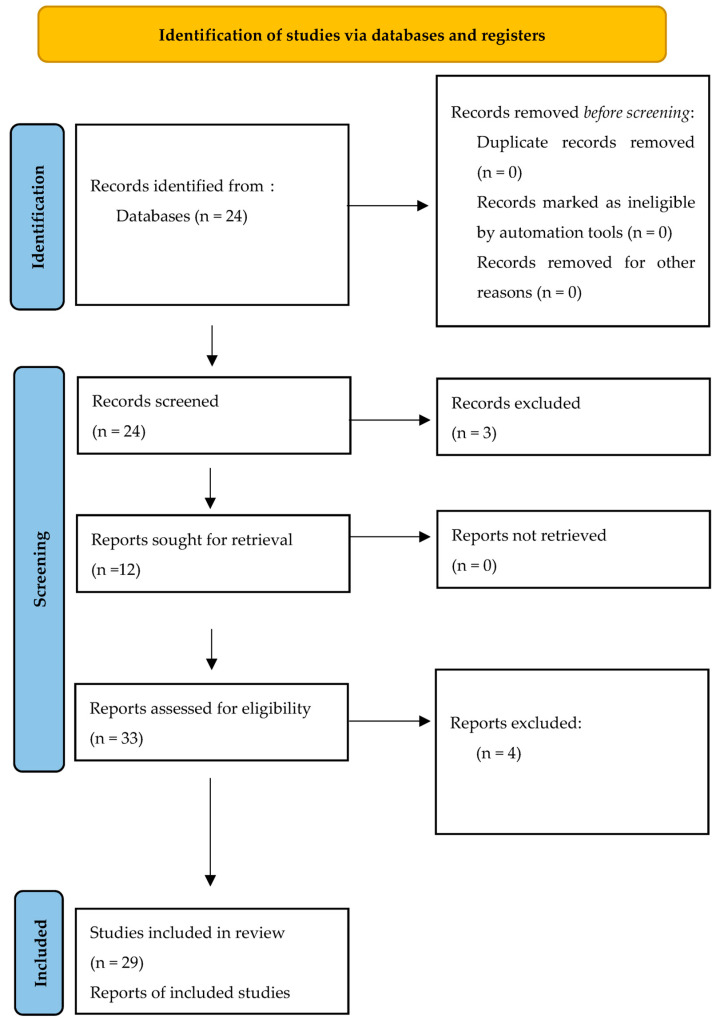
PRISMA flow diagram.

A total of twenty-four articles were identified in databases. After the titles and abstracts had been screened, three articles were excluded because they did not reach the eligibility criteria. From the analysis of these twenty-one articles and their references, twelve other studies were found eligible for inclusion. After a careful evaluation of the abstracts, titles and full texts aiming to assess whether the articles dealt with Carlevale lens and actually reported data about the implantation of this lens, surgical outcomes, visual and refractive results, twenty-nine studies were included (Figure 1).

#### 3.1.1. Surgical Technique

**Figure 2:**
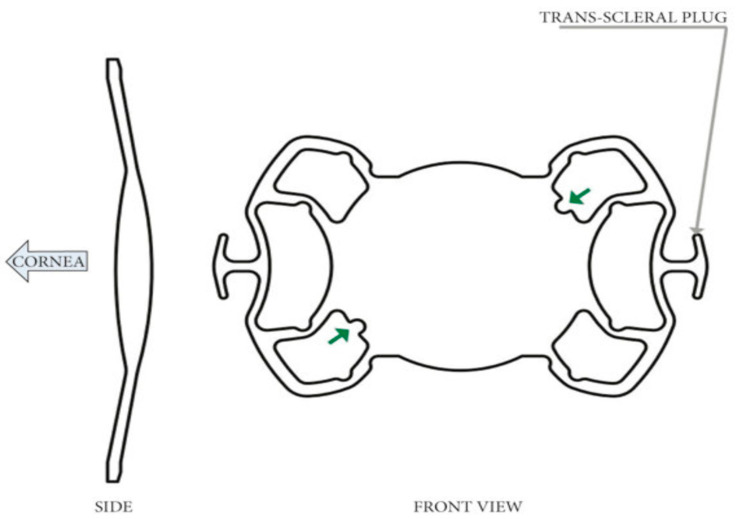
Design of Carlevale lens; green arrows indicate the specular incisions to identify the correct IOL position. Image sourced from the manufacturer’s website.

A Carlevale IOL is an acrylic one-piece lens with a total length of 13.2 mm and a 6.5 mm wide optic plate and an A-constant of 118.5 [20]. The 10° anterior angulation of the two haptics with respect to the optic plate avoids contact with the iris and should reduce the risk of a pupillary block [1]. The opposed T-shaped harpoons protruding off the closed haptics allow for self-anchoring to the sclera without sutures [1,21,22] (Figure 2).

The Carlevale IOL is foldable and injected via a dedicated cartridge and a disposable plunger injector through a 2.2 mm corneal incision [1]. Two small specular incisions in the haptics allow the surgeon to identify the correct IOL position, avoiding the upside-down injection.

The dioptric power ranges between −5 and +35 diopters and customized toric lenses are also available (cylinder power up to 10 diopters in steps of 1 diopter); the latter should always be placed along the horizontal axis of 0–180° [1,20,21].

The surgical technique consists of performing a conjunctival peritomy. In almost all cases it has to be performed nasally and temporally to create scleral tunnels along the 0–180° axis, usually at 3 o’clock and at 9 o’clock. When this was not allowed, or according to the surgeon’s preference, the scleral tunnels could be realized along another axis [20]. The scleral tunnels should be diametrically opposed by 180° [21,23,24]. For this reason, pre-marking the horizontal axis on the slit lamp before surgery has been suggested [25].

Two scleral flaps were sculpted by the surgeon with dimensions ranging between 3 × 3 and 4 × 4 mm [1,21,24,25,26,27,28,29]. With regards to sclerotomies, most of the studies preferred 25-gauge (G) sclerotomies at 1.5 to 2 mm from the limbus [1,9,21,23,25,27].

The other surgical option that emerged from literature was the creation of two scleral pockets for each side [28]; in this circumstance, almost all the authors were in agreement and preferred 23 G sclerotomies [8,9,21,22,30,31]. For each sclerotomy, Veronese et al. [23] proposed obtaining two self-sealing pockets by creating two lateral scleral tunnels of 1 mm perpendicularly to the incision [22]. In this way, the choice of using bigger sclerotomies could be justified as they allow for an easier externalization maneuver of the haptics from the scleral tunnels.

A corneal incision of less than 3 mm was then created to inject the Carlevale IOL using a cartridge. The correct loading of the IOL in the cartridge was dictated by two opposite incisions on the lens (supero-nasal and infero-temporal). When the first anchor was in the anterior chamber, the forceps on the other side took it and brought it out through the sclerotomy. Then, a second set of forceps was used to perform the same with the other anchor on the opposite side. At this time the IOL was “self-fixated” by the T-shaped anchors on the sclera under the flap. If the surgeon opted for scleral pockets instead, it was necessary to put the anchors into the pockets.

Regarding the haptics, 13/20 (65%) of the haptics were inserted through the ciliary sulcus, 4/20 (20%) were posterior to the ciliary body and 3/20 (15%) were inserted through the ciliary body [32]. The scleral plugs’ depth was investigated by anterior segment Optical Coherence Tomography (OCT) and was 247.20 ± 62.83 micron in the nasal sector and 265.50 ± 30.11 in the temporal one [32].

The most common formula for implantation was SRK T with an A-constant of 118.5 [8,20,22,23]. Other authors mentioned the use of SRK with an A-constant of 119.1 [21], Barrett Universal and Haigis [22], as well as SRKII with an A-constant of 118.5 [33]. Vaiano and colleagues used either Hoffer Q, Holladay 1 or the SRK/T formula and did not find statistically significant differences [34].

A crucial point of contention was whether to proceed with a pars plana vitrectomy (PPV). Based on the published evidence, it is suggested to perform a PPV every time [1,20,21,23,25,26,29,31,35,36].

Sidiropoulos and colleagues [22] preferred 25G PPV when there was a nucleus or an IOL in the vitreous cavity, while they used 27G PPV if a coexisting macular pathology occurred. The target was to create a complete posterior vitreous detachment and to prevent retinal complications. It was unclear if a three-ports approach was better than a five-ports one that was carried out by creating two sclerotomies for the fixation of the haptics [21,23,35]. According to Danese et al., 2021, this strategy reduces the invasiveness of the procedure [35]. On the other hand, three authors suggested using 25 G PPV, but only in presence of retinal comorbidities [8,9,22,31]. Fiore and colleagues suggested placing an infusion line in the anterior chamber at the 6 o’clock position to avoid intraocular hypotony if the surgery was limited to the anterior segment [8,9].

At the end of surgery, the most accredited strategy was to avoid scleral sutures [1,25,37], even if some authors recommended the use of Nylon 10.0 [21,25] or Vicryl 7.0 [28] to close the scleral flaps. The use of fibrin glue seemed not to be widespread [21]. In presence of scleral pockets, sutures should be avoided [23,35]. Only in myopic eyes was it advisable to perform scleral sutures, due to the major risk of scleral erosion [22]. Conversely, Fiore and colleagues, in two different manuscripts, suggested suturing the sclerotomies with a butterfly or cross-stitch point to immobilize the plug within the pockets in all the cases [8,9]. 

Finally, the conjunctiva can be closed with Vicryl 7.0 or 8.0 [1,8,9,22,25,27,29,37], but some authors preferred to avoid conjunctival sutures completely [23,35].

#### 3.1.2. Intraoperative Complications

Intraoperative complications turned out to be rare, and some authors reported no intra-surgery problems [22,23,24,25,28,32,35].

Most of the authors reported intraoperative complications related to the IOL, including damage to the optics or the anchors or the rupture of one T-shaped IOL harpoon during the injection or the scleral passage [1,9,21,29,30,38]. Another one, in terms of recurrence, was a mild vitreous hemorrhage [1,20,29].

In addition to these, IOL dislocation in the vitreous cavity was reported in three different works [8,21,29], which was probably related to a too quick injection of the Carlevale lens and/or the absence of an iris support [8,21]. Other sporadic intraoperative complications described were (i) mild vitreous bleeding from the iris or the ciliary body [21,29,38], (ii) IOL instability [8,38], (iii) sclerotomies-related iris trauma [20,29].

Moreover, in two patients (2.8%), Rouhette et al. described a reverse IOL implantation solved with the help of two micro-manipulators after IOL fixation to the sclera. In addition, Rossi and colleagues reported a case of corneal edema, a case of retinal rupture and a case of retinal detachment [1]. In the literature, there was only one study reporting a case of too thin scleral flaps that required a scleral patch [21]. Danese et al., 2021 [39] and Dyrda et al., 2022 [40] described a transient clouding of the Carlevale lens, with spontaneous resolution, during the implantation [39]. They conducted an in vitro exam which suggested that the opacification was due to thermal shock of the hydrophilic polymers, and it disappeared after temperature stabilization [40]. Intraoperative complications were easily fixed and did not lead to sequelae.

#### 3.1.3. Postoperative Complications

A vitreous hemorrhage [9,20,21,22,25,26,28,29,32] followed by a transient macular edema [1,8,9,21,22,24,25,28,29,31,32,41] was reported after surgery by most authors. Only two cases of chronic macular edema were observed but they were attributed to a pre-existing edema and to an ocular trauma. The use of non-steroidal anti-inflammatory drugs and/or corticosteroid led to recovery within several months [8,9].

Regarding intraocular pressure, no significant variation in the mean IOP was reported; some authors described a transient increase in the IOP in patients with pseudoexfoliation and in glaucoma patients [20,25,30], and other authors reported a reduction of the same parameters and a transient hypotony [8,21,22,28,31,38,41].

Barca et al., 2020, recorded two eyes (6.2%) with pigment dispersion and reverse pupillary block, solved by nd:YAG peripheral Iridotomy, and in one case, treatment with antiglaucoma eye drops [25].

In their case series, some authors described just one case of eyes with plugs located outside the scleral pockets and/or haptics exposure [1,24,29,36], while D’Agostino et al. [28] reported two cases of plug exposure. 

In addition, Rossi et al., 2021, reported two cases of retinal tears and two cases of retinal detachments [1]. One case of a late retinal detachment was described also by Vaiano and colleagues [41]. Five cases of transient corneal edema were reported by Rohuette et al. [21] and one by Franco et al. [24]. Two different studies referred to one case of corneal decompensation requiring DSAEK [42,43]. Finally, Seknazi et al., 2021, observed a case of a neurotrophic ulcer [26]. One case of endophthalmitis, one of IOL dislocation and three of IOL tilt were also described [29,30]. 

The surgical techniques and the intra and postoperative complications are recorded in Table S4 in the Supplementary Material.

#### 3.1.4. Visual Outcomes

All the authors reported an improvement in the best corrected visual acuity (BCVA) [1,20,21,22,24,25,30,32,34,38,44,45] (Table 1), and in comparison to the Artisan iris claw lens (Artisan Aphakia IOL model 205, Ophtec BV, Gro-ningen, The Netherlands), no significant difference (*p* = 0.19) was highlighted [26,29,36]. Some authors did not consider the refractive and/or visual outcomes [27,28,38,43] while other authors reported only post-operative visual data [8,9,26,40]. Moreover, Fiore and colleagues compared Carlevale IOL scleral fixation with 23-gauge to 25-gauge sclerotomies and did not show any significant differences in terms of BCVA between the two techniques [9].

Carlevale IOL showed good stability: the IOL tilt range from 2.08 ± 1.19 to 3.1 ± 1.1 degrees [1,24,25,41,46] measured by anterior segment optical Coherence Tomography (Table 2). Using Ultrasound Biomicroscopy (UBM) Mularoni and colleagues observed a mean vertical tilt of 0.19 ± 0.22 mm in the absence of a horizontal tilt.

Regarding corneal astigmatism (Table 3), some authors did not show any changes from the pre-operative to the postoperative time [21,28,36,44]. Mularoni and colleagues [32] reported a small increase between pre-operative and post-operative astigmatisms. Other authors documented a lower amount of astigmatism compared to Artisan iris claw and three-piece IOLs implantation [26,28,35,36]. Van Severen and colleagues reported an increase in postoperative astigmatism at one month of 0.47 ± 1.44 in Carlevale group versus 0.52 ± 1.47 in the Artisan Group [29]. Comparing the Carlevale IOL to the three-piece scleral-fixated IOL, D’Agostino et al. [28] concluded that there was no difference in mean postoperative astigmatism, but when a surgical astigmatism was present, it was significantly higher after three-piece IOL implantation (group with three-piece IOL: 1.91 ± 2.07, versus group with Carlevale: 0.67 ± 0.88; *p* = 0.04).

## 4. Discussion

Based on the existing literature, Carlevale IOL implantation seems to be a safe and effective scleral fixation technique. Together, the studies showed that BCVA improved after surgery, although with different methods and timing. The only exception was trauma, which showed a trend toward lower acuities and required a longer surgical time [1]. 

Carlevale IOL implantation also contributed to the determination of a significant separation between anterior and posterior segments [1,8,9,27], even if one case of a dexamethasone intravitreal implantation that migrated into the anterior chamber is mentioned in the literature [47].

Through our analysis of the intra- and post-operative complications we can affirm the good safety profile of this suture-less IOL, with a low rate of endophthalmitis, damage to corneal endothelial cells and IOL dislocation [8,35,48]. The few cases of scleral erosion that have been observed were not related to the lens but to a thinner sclera, such as in myopic eyes [49], and this complication was solved by rotating the lens and creating new scleral flaps [49]. Sutures do not guarantee the stability of the IOL [9], while 25G sclerotomies could allow a suture-less technique to be used without the risk of early hypotony [8]. Additionally, 27-gauge sclerotomies could minimize this risk [38]. Moreover, the Carlevale lens is suggested to be the better choice in cases of recurrent IOL dislocation [50] and in children-age patients [33].

Regarding pupillary block, although it emerged as an uncommon complication [25], a recent study by Schranz and colleagues suggests that it is a risk factor associated with scleral-fixated IOLs [51]. The analysis and comparison of different surgical techniques for scleral fixation and various types of lenses, including the Carlevale lens, indicate that all can potentially lead to cases of reverse pupillary block in the absence of an iridectomy.

According to our review, scleral flaps appear to be more manageable than pockets, with a lower risk of hypotony [21], even if scleral pockets were proposed to be simpler and to offer a better stability to the lens [9,22].

The innovative design of the Carlevale IOL, with the haptics angled anterior to the optic plate and with an overall length of 13.5 mm and a 6.5 mm large optic, seem to improve IOL centration [20,48]. This lens also reduced the risk of iris capture, and was more independent from anatomical variations [1,48,50]. The good self-centering of the IOL and its stability appeared to limit the development of macular edema [20]. 

Regarding lens tilting, most studies [1,24,25,32,41,46] assessed this parameter in two dimensions (horizontal and vertical planes). In contrast, Schranz and colleagues employed a 3D program for a more realistic evaluation of tilting, which revealed higher values. Based on this analysis, the authors concluded that there is a significant difference in tilting, measured as the angle in degrees between the visual axis of the eye and the optical axis of the lens, among patients undergoing scleral fixation with various types of lenses and techniques compared to the control group [51]. 

The post-operative evaluation of astigmatisms did not show differences from the pre-operative data. In this regard, a small corneal incision appears to induce less astigmatism than ACIOLs, iris claw lenses [26,36,52] or three-piece fixed-scleral IOLs [28]. 

From our analysis, no differences have been found in terms of visual and surgical outcomes between three-pieces scleral-fixation IOLs and Carlevale IOLs [24,28], with the advantages of the Carlevale IOLs of less induced astigmatism, less dislocation and easier and faster surgical time [28]. Carlevale IOLs also demonstrated a better refractive accuracy and induced less astigmatism compared to the Artisan lenses, with faster surgical time [26,36] and less postoperative complications.

Consequently, this surgical technique also appears to be feasible for anterior segment surgeons after adequate training [9,20,22,53], as we counted very few adverse retinal events, even when a vitrectomy was not performed [21,25]. 

To improve IOL placement and reduce the risk of the IOL dropping into the vitreous chamber, the corneal tunnel should be positioned opposite the sclerotomy site, especially in cases without iris support and with pupillary stupor [8,9].

The other critical step is the extraction of the fragile haptics, which is simplified by the distinctive design of the haptics of the Carlevale lens that allow self-centering of the IOL with minimal manipulation [25,26]. The strong fixation of the T-shaped caps seems to justify the good stability of the lens with a lower risk of tilt and an easier and faster surgical time [32,48]. Moreover, the similar outcomes obtained across several studies suggest a good reproducibility and standardization of this technique [53].

Based on this encouraging evidence, it is recommended that future research efforts focus on comparative studies to evaluate the efficacy and outcomes of scleral fixation using the Carlevale lens compared to established techniques. These studies should include longer follow up periods and use a standardized implantation procedure to ensure the consistency and reliability of the results. Such research would be valuable in establishing guidelines and protocols to enhance surgical outcomes and provide a more comprehensive understanding of the advantages and limitations associated with the Carlevale lens.

### Limitations

Scleral fixation with s Carlevale lens is a relatively recent technique; the main limitation of the present systematic review is the heterogeneity among the papers.

In addition, in this review, it was not possible to assess the quality of evidence by applying the GRADE (Grading of Recommendations Assessment, Development and Evaluation) system. 

Several authors employed anterior segment tomography to verify the correct position of the IOL; others just evaluated it through an anterior segment examination [1,20,21,22]. Only Mularoni and colleagues investigated the IOL tilt by performing a UBM examination [32]; moreover, there were no standard criteria to define “hypotony” and “ocular hypertension”, which were only specified by Sidiropoulos et al., 2022 [22] as less than or equal to 5 mmHg and more than or equal to 22 mmHg.

Most of all the examined studies had a small sample size and only four studies carried out a comparison with previously used IOLs [24,26,28,36]. More comparison studies with bigger sample sizes are needed to define the superiority of the Carlevale IOL.

The mean follow up time was six months, with differences across all of the studies. Longer follow up time would be desirable to better define post-operative complications such as scleral erosion, exposition of the haptics from the conjunctiva and glaucoma.

## 5. Conclusions

In conclusion, the published evidence indicates that Carlevale scleral fixation is a safe and effective technique. It seems to be a valid choice in the case of aphakia or in the case of the absence or instability of the capsular support thanks to the method of implantation, the few intraoperative complications, the self-centration and the good postoperative visual acuity and stability. It could be assumed that the standardization of the surgical technique may improve the surgical outcomes in terms of IOL positioning, centering and visual acuity.

## Figures and Tables

**Table 1:** Pre and post-operative BCVA and Follow-up. (In the table only the studies that mentioned pre and post-operative data were presented).

Authors	Sample	Pre-Operative BCVA	1 Months Visual Outcome	3 Months Visual Outcome	6 Months Visual Outcome	12 Months Visual Outcome	Post-Operative BCVA	Follow Up Times
Danese et al., 2022 [35]	3	-20/40 -Counting Fingers -20/50	-	-	-	-	20/20 20/400 20/32	8 months 8 months 5 months
Danese et al., 2021 [39]	1	20/200	-	-	-	-	20/25	3 months
Danese et al., 2024 [44]	35	0.9 ± 0.6 logMAR	-	-	-	-	0.5 ± 0.5 logMAR	24.5 ± 16.9 months (MD ± DS)
Vaiano et al., 2021 [41]	54	0.93 ± 0.61 logMAR	-	0.42 ± 0.34 logMAR	0.42 ± 0.37 logMAR	0.38 ± 0.38 logMAR	-	-
Gotzaridis et al., 2021 [46]	5	0.9 ± 0.7 logMAR	-	-	-	-	0.26 ± 0.32 logMAR	7 months (Median)
Rohuette et al., 2021 [21]	72	3.2/10 ± 3.1	-	-	7.2/10 ± 2.1	-	-	-
Gabai et al., 2021 [30]	13	0.75 ± 0.5 logMAR	-	-	-	-	0.28 ± 0.3	7.5 ± 7 (MD ± DS)
Georgolas et al., 2022 [20]	169	0.58 ± 0.49	-	-	-	-	0.09 ± 0.1 LogMAR	9 months (Median)
Rossi et al., 2021 [1]	78	0.86 ± 0.56 logMAR	-	-	-	-	-	-
Veronese et al., 2020 [23]	4	0.50 ± 0.33 logMAR	-	-	-	-	0.08 ± 0.08 logMAR	6.50 ± 1.29 months (MD ± DS)
Barca et al., 2020 [25]	32	0.46 ± 0.29 logMAR;	-	0.22 ± 0.18 logMAR (4M)	-	0.13 ± 12 logMAR (8M)	-	8.15 (Mean)
Sidiropulos et al., 2022 [22]	27	0.85 ± 0.59 logMAR;	-	-	0.36 ± 0.34 logMAR	0.35 ± 0.32 logMAR	-	Not Applicable
Caporossi et al., 2021 [31]	60	0.46 ± 0.60 logMAR;	-	-	-	-	0.36 ± 0.51 logMAR (4M)	4 Months
Januschowski et al., 2021 [38]	16	0.83 ± 0.8 logMAR	-	-	-	-	0.65 ± 0.7 logMAR	2.2 ± 1.7 (MD ± DS)
Kymionis et al., 2020 [43]	1	Hand Movement	-	-	-	-	-	6 months
Petrelli et al., 2020 [45]		Hand Movement	-	-	-	-	0.3 (decimal scale)	Up to 4M
Franco et al., 2022 [24]	28	0.78 logMAR	-	0.23 logMAR	0.23 logMAR	0.23 logMAR	-	-
Ananikas et al., 2022 [33]	1	Counting Fingers	-	-		-	0.17 (decimal scale)	3 years
Vaiano et al., 2023 [42]	10	1.78 ± 0.76	-	1.01 ± 0.59	0.71 ± 0.37	0.525 ± 0.30	-	-
Bodin et al., 2022 [36]	25	0.70 ± 0.52 logMAR	-	0.33 ± 0.35	-	-	-	-
Mularoni et al., 2021 [32]	10	0.37 ± 0.33 logMAR	-	-	-	-	0.09 ± 0.08 logMAR	8.70 ± 4.16 (MD ± DS)
Van Severen et al., 2023 [29]	85	0.71 ± 0.67 logMAR (n = 85)	0.29 ± 0.75 logMAR (n = 82)	-	-	-	0.29 ± 0.75 logMAR (n = 82)	1 months

**Table 2:** IOLs’ tilting. (In the table Only the studies that mentioned IOL’s tilting data are presented in the table).

Author	Sample (N° Eyes)	Median Follow-Up Time (Months)	Mean IOL Tilt
Fiore et al., 2021 [8]	18	11.2 ± 4.1	2.2° ± 1.6°
Vaiano et al., 2021 [41]	54	12	3.1 ± 1.1°
Gotzaridis et al., 2021 [46]	5	9	2.1 ± 1.9°
Barca et al., 2020 [25]	32	8	2.08 ± 1.19°
Mularoni et al., 2021 [32]	10	8.70 ± 4.16	Mean vertical tilt 0.19 ± 0.22 mm; mean horizontal tilt 0.05 ± 0.03 mm
Franco et al., 2022 [24]	28	12	2.75°

**Table 3:** Surgically induced astigmatism. (Only the studies that mentioned surgical induced astigmatism data are presented in the table).

Author	Sample	Preoperative Astigmatism (D) MD ± DS	Postoperative Astigmatism (D) MD ± DS	Surgical Induced Astigmatism SIA (D)	Median Follow-Up Time (Months)
Rohuette et al., 2021 [21]	72	1.4 ± 2.3	1.35 ± 0.9	-	6
Bodin et al., 2022 [36]	25	1.90 ± 1.65	1.96 ± 1.48	-	3
Mularoni et al., 2021 [32]	10	−1.20 ± 0.85	−0.90 ± 0.57	-	8.70 ± 4.16
D’agostino et al., 2021 [28]	16	0.46 ± 0.96	0.67 ± 0.88 (n = 7)	-	6
Seknazi et al., 2021 [26]	20	-	-	0.72 ± 0.52	6.42 ± 3.96
Van Severen et al., 2023 [29]	79	−1.16 ± 1.34	-	0.47 ± 1.44	1
Danese et al., 2024 [44]	35	−0.1 ± 0.8	−0.9 ± 1.9 D	-	24.5 ± 16.9

## Supplementary Materials

The following supporting information can be downloaded at: https://www.mdpi.com/article/10.3390/jcm13113287/s1, Table S1: Newcastle–Ottawa Scale for Critical Appraisal for Studies; Table S2: Quality assessment of the included case series using the Joanna Briggs Institute (JBI) Critical Appraisal Checklist for Case Series; Table S3: Quality assessment of the included case series using the Joanna Briggs Institute (JBI) Critical Appraisal Checklist for Case Reports. Table S4. This table shows data relating to surgical technique, intra and post-operative complications of each examined study. IOL: intraocular lens; DSAEK: Descemet’s Stripping Automated Endothelial Keratoplasty; UTDSAEK: Ultra Thin Descemet’s Stripping Automated Endothelial Keratoplasty).
