# Supplementary material for: Current Evidence for a New Surgical Technique for Scleral Fixation: The Implantation of a Carlevale Lens, a Systematic Review
Source: J Clin Med. 2024 Jun 3;13(11):3287. doi: 10.3390/jcm13113287 (PMC11172714; doi:10.3390/jcm13113287)
Supplement: Supplementary file 1 [file jcm-13-03287-s001.zip › jcm-2981148-supplementary.pdf]

Journal of
Clinical Medicine

**Table S1.** Newcastle–Ottawa Scale for Critical Appraisal for Studies.

| | | Selection | | | | Comparability | Outcome | | | |
|---|---|---|---|---|---|---|---|---|---|---|
| **Source** | **Study design** | **Representativeness of the exposed cohort** | **Selection of the non-exposed cohort** | **Ascertainment of exposure** | **Outcome was not present at start** | **Based on design and analysis** | **Assessment of outcome** | **Follow up long enough** | **Adequacy of follow up** | **Total score** |
| **Fiore et al, 2021 [9]** | Longitudinal | 1(b) | 1(a) | 1(a) | 1(a) | 1(a) | 1(b) | 1(a) | 1(b) | 8/9 |
| **Bodin et al, 2022 [36]** | Longitudinal | 1(b) | 1(a) | 1(a) | 1(a) | 1(a) | 1(b) | 1(a) | 1(b) | 8/9 |
| **Seknazi et al, 2021 [16]** | Longitudinal | 1(b) | 1(a) | 1(a) | 1(a) | 1(a) | 1(b) | 1(a) | 1(b) | 8/9 |
| **Franco et al, 2022 [14]** | Longitudinal | 1(b) | 1(a) | 1(a) | 1(a) | 1(a) | 1(b) | 1(a) | 1(b) | 8/9 |
| **D'Agostino et al, 2021 [18]** | Longitudinal | 1(b) | 1(a) | 1(a) | 1(a) | 1(a) | 1(b) | 1(a) | 1(b) | 8/9 |
| **Van Severen et al, 2023 [29]** | Longitudinal | 1(b) | 1(a) | 1(a) | 1(a) | 1(a) | 1(b) | 1(a) | 1(b) | 8/9 |

**Table S2.** Quality assessment of the included case series using the Joanna Briggs Institute (JBI) Critical Appraisal Checklist for Case Series.

| Source | Were there clear criteria for inclusion in the case series? | Was the condition measured in a standard, reliable way for all participants included in the case series? | Were valid methods used for identification of the condition for all participants included in the case series? | Did the case series have consecutive inclusion of participants? | Did the case series have complete inclusion of participants? | Was there clear reporting of the demographics of the participants in the study? | Was there clear reporting of clinical information of the participants? | Were the outcomes or follow up results of cases clearly reported? | Was there clear reporting of the presenting site(s)/clinic(s) demographic information? | Was statistical analysis appropriate? |
|---|---|---|---|---|---|---|---|---|---|---|
| Barca et al, 2020 [15] | yes | yes | yes | no | yes | yes | yes | yes | yes | yes |
| Veronese et al, 2020 [13] | yes | yes | yes | no | yes | yes | yes | yes | yes | yes |
| Rossi et al, 2021 [1] | yes | yes | yes | no | yes | yes | yes | yes | yes | yes |
| Vaiano et al, 2021 [41] | yes | yes | yes | yes | yes | yes | yes | yes | yes | yes |
| Vaiano et al, 2021 [42] | yes | yes | yes | no | Unclear | unclear | unclear | yes | yes | yes |
| Vaiano et al, 2021 [33] | yes | yes | yes | yes | yes | no | no | yes | yes | yes |
| Gotzaridis et al, 2021 [46] | yes | Yes | yes | no | yes | yes | yes | yes | yes | yes |
| Mularoni et al, 2021 [32] | yes | yes | yes | no | yes | yes | yes | yes | yes | yes |
| Januschowski et al, 2021 [38] | yes | yes | yes | no | yes | no | no | yes | no | yes |
| Rouhette et al, 2021 [21] | yes | yes | yes | no | yes | yes | yes | yes | yes | yes |
| Danese et al, 2021 [39] | yes | yes | yes | no | yes | no | no | no | no | no |
| Caporossi et al, 2021 [31] | yes | yes | yes | no | yes | no | no | yes | no | yes |
| Fiore et al, 2021 2 | yes | yes | yes | no | yes | yes | yes | yes | yes | yes |
| Gabai et al, 2022 [30] | yes | yes | yes | no | yes | no | no | yes | no | yes |
| Georgalas et al, 2022 [11] | yes | yes | yes | no | yes | yes | yes | yes | yes | yes |
| Sidiropoulos et al, 2022 [12] | yes | yes | yes | no | yes | yes | yes | yes | yes | yes |
| Danese et al, 2023 [44] | yes | yes | yes | no | yes | yes | yes | yes | yes | yes |

**Table S3.** Quality assessment of the included case series using the Joanna Briggs Institute (JBI) Critical Appraisal Checklist for Case Reports.

| Source | Were patient's demographic characteristics clearly described? | Was the patients' history clearly described and presented as a timeline? | was the current clinical condition of the patient on presentation clearly described? | Were diagnostic tests or assessment methods and the results clearly described? | Was the intervention(s) or treatment procedure(s) clearly described? | Was the post-intervention clinical condition clearly described? | Were adverse events (harms) or unanticipated events identified and described? | Does the case report provide takeaway lessons? |
|---|---|---|---|---|---|---|---|---|
| De Angelis et al, 2023 [37] | yes | yes | yes | yes | yes | yes | no | yes |
| Danese et al, 2022 [35] | yes | yes | yes | yes | yes | yes | no | yes |
| Petrelli et al, 2020 [45] | yes | yes | yes | yes | yes | yes | no | yes |
| Kymionis et al, 2020 [43] | yes | yes | yes | yes | yes | yes | yes | yes |
| Ananikas et al, 2022 [33] | yes | yes | yes | yes | yes | yes | no | yes |
| Dyrda et al, 2022 [40] | yes | no | yes | yes | yes | yes | yes | yes |

**Table S4.** This table shows data relating to surgical technique, intra and post-operative complications of each examined study. IOL: introcular lens; DSAEK: Descemet's Stripping Automated Endothelial Keratoplasty; UTDSAEK: Ultra Thin Descemet's Stripping Automated Endothelial Keratoplasty.)

| Author | Sample (eyes) | Technique | Intraoperative Complications | Postoperative Complications |
|---|---|---|---|---|
| Fiore et al, 2021 [8] | 18 | Scleral pockets;<br>25Gauge Vitrectomy performed in case of coexisting retinal pathologies or intraoperative complications (n=7). | - Plugs externalized with a hand-shake-like technique (n=1)<br>- IOL dropped in vitreous chamber (n=1) | - Chronic macula edema (not resolved) (n=2) |
| Fiore et al, 2021 [9] | 32<br>(Group 1 n=23; Group 2 n=9) | Group 1 plugs were externalized through a 23G sclerotomy and placed within 2 scleral pockets<br>Group 2, plugs were externalized through a 25G sclerotomy and covered by 2 scleral flaps. | Group 1:<br>- rupture of one tip of the plug (n=3)<br>Group 2:<br>- none | Group 1:<br>- Transient Macular edema (n=1)<br>- Chronic Macular edema (n=1)<br>- Vitreous Hemorrhage |
| Rohuette et al, 2021 [21] | 72 | Scleral plugs or scleral pockets;<br>all patients underwent complete 23 or 25Gauge vitrectomy | - damage of the optics / anchors or rupture of T shaped harpoons (n=9, 12,5%)<br>- reverse implantation (n=2, 2,8%)<br>- iol dropped in vitreous chamber (n=4, 5,6%)<br>- too thin scleral patch needing a scleral patch (n=1)<br>- bleeding from iris (n=1) and ciliary body (n=1) | - transient vitreous hemorrhage (12% after 1 week; no cases at 3 and 6 months)<br>- transient macular edema (2,8% at 3 months; resolved at 6 months)<br>- retinal detachment (1,4% after 3 months)<br>- corneal edema (5% after 1 week; 1,4% after 3 and 6 months)<br>- transient hypotony(30% after 1 week; no cases after 3 and 6 months) |
| Gabai et al, 2021 [30] | 13 | Scleral pockets; all patients underwent 25Gauge vitrectomy; complete vitrectomy when necessary (n=2) | - Rupture of IOL haptic (n=1, 7,7%) | - Transient vitreous hemorrhage (n=1, 7,7%)<br>- Transient increase of IOP (n=2, 15,4%) |

| | | | | |
|---|---|---|---|---|
| | | | | - Mild endophtalmitis (n=1, 7,7%) |
| Georgolas et al, 2022 [20] | 169 | Scleral flaps; all patients underwent 3-port pars plana 23 or 25 Gauge vitrectomy | - mild vitreous hemorrhage (n=12, 7,1%)<br>- sclerotomies-related iris trauma (n=5, 2,5%) | - vitreous hemorrhage (n=8, 4,7%; resolved within 3 weeks)<br>- transient increase of IOP(n=28, 16,5%) |
| Rossi et al, 2021 [1] | 78 | Trans-scleral plugs;<br>all patients underwent 3-port complete vitrectomy | - rupture of T-shaped harpoons (n=1, 1,3%)<br>- vitreous hemorrhage (n=2, 2,5%)<br>- corneal edema (n=2, 2,5%)<br>- retinal tears (n=2, 2,5%)<br>- retinal detachment (n=1, 1,3%) | - cystoid macular edema (n=4, 5,1%)<br>- vitreous hemorrhage (n=1, 1,3%)<br>- ocular hypertension n=2, 2,5%)<br>- plugs located outside scleral pockets or erosion requiring patch (n=1, 1,3%)<br>- retinal tears n=2, 2,5%)<br>- and retinal detachment n=2, 2,5%)<br>- bullous keratopathy requiring DSAEK (n=1, 1,3%) |
| Bodin et al, 2022 [36] | 25Carlevale vs 37Artisan | Scleral pocktes or flaps;<br>All patients underwent 25 Gauge vitrectomy | - -rupture of the haptics (n=1) | - Transient vitreous hemorrhage(n=1)<br>- ocular hypotonia with choroidal detachment (n=1)<br>- plugs located outside scleral pockets or erosion (n=1) |
| Januschowsky et al, 2021 [38] | 17 | Scleral flaps;<br>27 Gauge vitrectomy was performed in case of complications | - No intraoperative complications | - anterior chamber and vitreous hemorrhage with tilted IOL (n=1) |

| | | | | |
|---|---|---|---|---|
| | | | | - -sectoral iris root defect (n=1)<br>- macular edema (n=1)<br>- ocular hypotonia (n=7, 44%) (in 2 cases need addictional or adjustive sutures) |
| Vaiano et al, 2021 [41]<br>" | 54 | Scleral flaps;<br>in the majority of cases 25Gauge vitrectomy was performed | - rupture of the IOL haptics (n=6, 11%) | Early:<br>- transient corneal edema (n=5,9,25%)<br>- infiammatory response in anterior chamber (n=4,7,4%)<br>- mild intraocular hemorrage (n=4,7,4%)<br>- -ocular hypertension (n=2, 3,7%)<br>Late:<br>- Macular edema (n=4,7,4%)<br>- Haptics exposure (n=2, 3,7%)<br>- Retinal detachment (n=1, 1,85%) and epiretinal membrane (n=1, 1,85%) |
| Vaiano et al, 2021 [34] | 25 | Scleral flaps;<br>in the majority of cases 25 Gauge vitrectomy was performed | Not Applicable | Not Applicable |
| Vaiano et al, 2023 [42] | 10 | UT-DSAEK combined with Carlevale's sutureless scleral fixation IOL | Not Applicable | - Transient macular edema (n=1)<br>- Transient IOP increase (n=3) |
| Barca et al, 2020 [25] | 32 | Scleral flaps (n=20) or pockets (n=12); 25 or 27G vitrectomy was performed in all cases. | Not Applicable | - Transient vitreous hemorrhage (n=1, 3,1%)<br>- Transient cystoid macular edema (n=1, 3,1%) |

| | | | | |
|---|---|---|---|---|
| | | | | - Intraocular hypertension (n=1, 3,1%)<br>- Reverse pupillary block (n=2, 6,1%) |
| Sidiropulos et al, 2022 [22] | 27 | Scleral pockets; 25 or 27 Gauge vitrectomy was performed when necessary (PPV group n=8) | Not Applicable | Not PPV Group:<br>- Transient vitreous hemorrhage (n=2, 7,4%)<br>- cystoid macular edema (n=1, 3,7%)<br>- transient pcular hypertony (n=1, 3,7%)<br>- transient ocular hypotony (n=2, 7,4%)<br>-<br>PPV Group:<br>-ocular hypotony (n=1, 12,5%) |
| Caporossi et al, 2021 [31] | 60 | All patients underwent 23 or 25 Gauge Vitrectomy | Not Applicable | - Vitreous hemorrhage (1)<br>- Transient macular edema (3)<br>- Transient reocular hypotony (n=4) |
| Mularoni et al, 2021 [32] | 10 | Scleral flaps | No intraoperative complications | - Transient vitreous hemorrhage (n=1)<br>- Cystopid macular edema (n=1)<br>- Epiretinal membrane (n=1) |
| Kymionis et al, 2020 [43] | 1 | Implantation of a SSF posterior chamber IOL with 23G sclerotomy combined with DSAEK for the management of IOL dislocation and corneal endothelial decompensation; scleral pockets | Not Applicable | - Vitreous hemorrhage (n=1) |

| | | | | |
|---|---|---|---|---|
| Seknazi et al, 2021 [26] | 20<br>(Group 1: Artisan n=22;<br>Group 2: Carlevale n=20) | Scleral flaps; all patients underwent complete vitrectomy | -Rupture of the plug (n=1, 5%) | - Transient vitreous hemorrhage (n=1, 5%)<br>- Cystoid macular edema (n=2, 10%)<br>- Neurotrophic ulcer (n=1, 5%) |
| Franco et al, 2022 [24] | 28<br>(Group 1:Carlevale n=28;<br>Group 2:Three pieces lens n=25) | Scleral flaps | Not Applicable | - Macular edema (n=2, 7,1%)<br>- Haptic exposure (n=1, 3,6%)<br>- Transient corneal edema (n=1, 3,6%)<br>- Bullous keratopathy requiring DSAEK (n=1, 3,6%) |
| D'agostino et al, 2021 [28] | 16<br>(Group 1: Three pieces lens n=15;<br>Group 2: Carlevale n=16) | Trans-scleral plugs<br>were left over the bare sclera (n=9) or scleral flaps (n=7) | Not Applicable | - Macular edema (n=7, 43,7%)<br>- Ocular hypotony (n=1, 6%)<br>- Among the eyes with plugs left over bare sclera, 1 developed bleb and 2 plug exposure |
| Danese et al, 2021 [39] | 1 | 25 Gauge vitrectomy | Transient clouding of the Carlevale lens | - Not Applicable |
| Danese et al, 2021 [35] | 3 | Combined IOL explantation, posterior vitrectomy and secondary Carlevale implantation | Not Applicable | - No postoperative complications reported |
| Danese et al, 2023 [44] | 35 | Sub-conjunctival<br>positioning of the anchors without flaps. When necessary, 25Gauge vitrectomy was performed | -partial rupture of the anchor (n=1) | - No postoperative complications reported |
| Dyrda et al, 2022 [40] | 1 | Not Applicable | Transient clouding of the Carlevale lens | - Not Applicable |

| | | | | |
|---|---|---|---|---|
| Van Severen et al, 2023 [29] | 101<br>(Group 1:Carlevale n=101;<br>Group 2: Artisan n= 77) | Scleral flaps; all patients underwent 25Guage vitrectomy | Intraocular hemorrhage: in anterior chamber (n=2) and in vitreous cavity(n=1)<br>- Haptic slipped out of the sclerotomy(n=1, 1%)<br>-haptic broken (n=2, 2%)<br>-Iridodialysis (n=1, 1%) | - Cystoid macular edema (n=15, 14,9%)<br>- Persistent corneal edema (n=14, 13,9%)<br>- Bleeding in anterior chamber or vitreous cavity (n=9, 8,9%)<br>- IOL tilt (n=3, 3%)<br>- Extrusion of the haptic (n=1, 1%) |
| Ananikas et al, 2022 [33] | 1 | Scleral tunnels; 25Gauge 3-port vitrectomy | Not Applicable | No postoperative complications |
| Petrelli et al, 2020 [45] | 1 | Combined penetrating keratoplasty with implantation of Carlevale IOL; scleral pockets | Not Applicable | No postoperative complications |
| De Angelis et al, 2022 [37] | 2 | Combined penetrating keratoplasty with implantation of Carlevale IOL; scleral pockets | Not Applicable | Not Applicable |
| Veronese et al, 2020 [23] | 4 | Plugs located under conjunctiva; all patients underwent 25 Gauge vitrectomy | Not Applicable | No postoperative complications |
| Gotzaridis,et al, 2021 [46] | 5 | Scleral flaps; all patient underwent 23 or 25 Gauge Vitrectomy | Mild vitreous hemorrhage form sclerotomies(n=1) | No postoperative complications |
